# Genomics of adaptation and wood properties in *Populus trichocarpa*

**DOI:** 10.1186/1753-6561-5-S7-O2

**Published:** 2011-09-13

**Authors:** Carl Douglas

**Affiliations:** 1 University of British Columbia, USA

## 

*Populus trichocarpa* (black cottonwood) and *P. balsamifera* (balsam poplar) are native to North America where large natural populations exist. *P. trichocarpa* has an extensive north-south range along the west coast, while *P. balsamifera* has a northerly range across the continent from Alaska to the North American east coast. Both species are well suited for highly productive bioenergy biomass plantations in north-temperate climates. We are investigating the large reservoirs of natural genotypic and phenotypic variation in wild populations of these species, with an initial focus on *P. trichocarpa*, to identify allelic variation underlying optimal biofuel and biomass traits that could be used for accelerated domestication. We sampled genetic variation in *P. trichocarpa* by Illumina transcriptome resequencing of 20 individuals from provenances along a latitudinal gradient from 60?N to 44?N. This analysis revealed extensive nucleotide, gene expression and alternative splicing polymorphism in 10,000 xylem-expressed genes. Alignment of transcript sequences to the *P. trichocarpa* Nisqually-1 reference genome was used to identify over 500,000 SNPs. These SNPs were combined with a larger SNP set generated by whole genome resequencing of multiple *P. trichocarpa* individuals carried out by the US DOE Bioenergy Sciences Center (BESC) to populate a *P. trichocarpa* SNP database. Using the Illumina iSelect process, we generated an Illumina Infinium bead array for genotyping of 34,000 SNPs that tag 3,700 candidate genes selected for their potential involvement in biomass, adaptation, and wood traits. Using this bead array in collaboration with BESC, we obtained candidate gene SNP genotypes for over 700 *P. trichocarpa* individuals grown in common gardens. This analysis of these data revealed extensive genotypic polymorphism in the targeted gene set. In parallel, we carried out phenotyping of the trees from the wild populations grown in common gardens, quantifying variation in over 85 morphological, growth, physiological, wood chemistry, and wood quality traits. Population genetic analysis of the SNP data, its use for genotype-phenotype association studies to identify candidate gene alleles underlying variation in traits of interest, and the prospects of using the results for marker assisted breeding will be discussed.

